# The impact of imaginary future generations on the preference for carbon tax schemes

**DOI:** 10.1371/journal.pone.0346904

**Published:** 2026-04-10

**Authors:** Yen-Lien Kuo, Wen-Chin Wu, Daigee Shaw, Bin-Tzong Chie, Yu-Tzung Chang, Yi-Lun Chuang, Yen-Ling Liu, Chun-Ta Fan

**Affiliations:** 1 Department of Economics, National Cheng Kung University, Tainan City, Taiwan; 2 Institute of Political Science, Academia Sinica, Taipei, Taiwan; 3 Institute of Economics, Academia Sinica, Taipei, Taiwan; 4 Department of Industrial Economics, Tamkang University, Taipei, Taiwan; 5 Department of Political Science, National Taiwan University, Taipei, Taiwan; Asian Development Bank Institute, JAPAN

## Abstract

Carbon pricing instruments have been found to be an effective incentive to mitigate climate change, but that surely increases the burden on the current generation. Some previous experiments found that people will have fewer or delayed gains after imagining the future. This research employed an experimental survey with a randomized treatment to investigate whether introducing imaginary future generations (IFGs) increases respondents’ probability of choosing carbon tax schemes. The survey was conducted at the end of 2021, collected 1,100 responses, with IFGs randomly introduced to half of the participants. Five carbon reduction schemes and their environmental, social, and economic consequences were presented to the respondents. These schemes include four hypothetical carbon tax schemes and a feed-in-tariff (FIT) scheme that is currently implemented in Taiwan as a comparator. Those carbon tax schemes can reduce carbon emissions by 50% from 2005 levels by 2050. This contrasts sharply with a no-tax scenario, which is projected to see emissions increase by 41%. The results showed that the carbon tax bundled with reduced VAT rates and lump-sum rebates was the most popular scheme, being the top choice for 35% of respondents. This appeal is likely attributable to its revenue recycling design, as well as its perceived superior social impacts compared to other schemes, which would increase the annual income of the lowest 20% income group by 7.5%. Moreover, the introduction of IFGs does significantly increase the probability by 11% that a respondent chooses the carbon tax scheme over the non-carbon tax scheme. The IFGs experience influences a broad demographic, including most respondents and wealthier individuals, rather than solely environmentalists, making them feel the social pressure of climate change concerns and be willing to mitigate it.

## 1 Introduction

The Sixth Assessment Report of the IPCC indicated that “climate change including increases in frequency and intensity of extremes have reduced food and water security, hindering efforts to meet Sustainable Development Goals (high confidence);” and that “changes are irreversible on centennial to millennial time scales in global ocean temperature (very high confidence), deep ocean acidification (very high confidence) and de-oxygenation (medium confidence)” [1]. The carbon dioxide might linger in the atmosphere for up to two hundred years [2]. Carbon dioxide emitted in the past and at present will affect the climate and environment that future generations will inherit. While carbon pricing instruments, such as a carbon tax, have been identified as a key tool for incentivizing emissions reduction [3], they often face significant public opposition due to the immediate costs of a carbon tax [4]. This presents a challenge: how to balance the present-day costs of carbon mitigation with the long-term benefits of a stable climate.

Addressing the environmental challenge requires innovative behavioral approaches that can bridge the psychological gap between present and future [5]. Saijo [6] argued that optimism as a characteristic of human nature would not help handle long-term problems, so he proposed “future design” as a framework to incorporate the preferences of future generations in its decision-making process. Not only was the future to be predicted, but also designing for the future should take place through negotiations with the imaginary future generations (IFGs), so that the rights of future generations would be respected. The studies have demonstrated that introducing IFGs can effectively improve sustainable resource allocation in ISDG experiments [7,8]. The research question of this study is how the IFG treatment promotes support for carbon taxes that increase the burden on the current generation and the benefits for future generations.

As advocated by Thaler and Sunstein [9], nudging is a form of choice architecture designed to help individuals make decisions that better align with societal goals, particularly by framing choices to highlight long-term benefits and thus overcoming common cognitive biases such as present bias. Hummel, D., & Maedche, A. [10] mentioned two choice architecture tools: structuring the choice task and describing the choice option, which address the idea of what and how to present to decision-makers, respectively. Additionally, the tools of choice architecture can be broken down into ten categories [11,12]. While defaults and social norms are frequently used, other measures are less common, including change effort, warnings/graphics, reminders, simplifications, disclosures, precommitment strategies, eliciting implementation intentions, and feedback. The most relevant example of climate change mitigation is promoting energy conservation by reporting comparative consumption and invoking social norms among close peers (social norms) [13]. Although nudges have been widely applied to encourage behavioral change, no studies have explored whether nudges can foster support for public policies that explicitly increase the financial burden of current generations, such as carbon taxes. In this study, we apply the concept of nudges by framing choices to highlight the environmental, economic, social impacts, and personal cost of carbon tax schemes.

As to responses to climate change (the biggest sustainability obstacle), it has not been proven that IFGs could be used to persuade the respondents to opt for carbon taxes (one of the climate change mitigation responses). The efficacy of Imaginary Future Generations (IFG) as a tool for sustainable decision-making connects with psychological research on ‘perspective-taking’ [14]. IFG may function as a form of perspective-taking that facilitates a “self-other overlap.” By imagining the lives of future generations, contemporary decision-makers mentally project themselves into the future to evaluate present decisions [15]. The IFG intervention invites individuals to mentally simulate the needs, welfare, and risks that people living in the future may face. It shifts attention from immediate personal costs to intergenerational consequences, thereby activating long-term prosocial motivation. Thus, our first hypothesis is that respondents who experience IFGs will become more willing to support carbon tax policies that aim to protect the welfare of future generations. Since the carbon tax is a financial burden for individuals, Hypothesis 2 is that the IFG effect will be stronger for wealthier people. It will also be examined in this paper.

In addition, this study reveals the most supported carbon tax scheme in Taiwan. In the next section, we review relevant literature. The third section introduces our research design, followed by empirical analysis. The final section concludes. This study is novel in applying the IFG to a concrete climate policy context, which may provide an essential strategy for overcoming the public opposition to carbon pricing.

## 2  Literature review

In order to examine whether IFGs can be a kind of behavioral intervention for carbon pricing, the key studies on the public support for carbon pricing, behavioral interventions for sustainability, and future design for pro-environmental behaviors will be discussed in this section.

### 2.1  Carbon pricing and public support

On 22 July 2024, the daily global average temperature reached a new record high. The UN Secretary-General called for action to limit temperature rise to 1.5°C by phasing out fossil fuels and scaling up investment in renewable energy. The Sixth Assessment Report of IPCC summarized that “economic instruments have been effective in reducing emissions,” and that “carbon pricing instruments have incentivized low-cost emissions reduction measures” [3]. However, citizens usually do not like to pay taxes; they might be concerned about the economic burdens. Equity and distributional impacts of such carbon pricing instruments can be addressed by using revenue from carbon taxes or emissions trading to support low-income households, among other approaches [3]. As a result, a well-designed carbon tax policy is essential to increase public acceptance, such as transparent revenue recycling (e.g., earmarking tax revenues, tax rebates, reducing VAT rates, or lump-sum transfers) to achieve revenue-neutral designs and improve fairness [4,16].

In Taiwan, a recent survey indicates a substantial reluctance to bear personal costs associated with carbon mitigation [17]. In 2021, 45% of Taiwanese respondents were unwilling to pay higher fuel taxes for environmental protection, and among those who were willing, only 17.6% would accept an increase of more than 6%. In contrast, 87% of respondents support government subsidies for purchasing electric scooters. This pattern illustrates a structural asymmetry in public preferences: Taiwanese citizens are generally supportive of government-funded incentives, yet they are reluctant to endorse policies that require direct individual payment. Such preferences shift the financial burden of mitigation away from current taxpayers and toward the government, ultimately pushing the costs onto future generations.

### 2.2 Future design for pro-environmental behaviors

To address sustainability concerns across generations, such as climate change, Future Design (FD) proposed by Saijo [18] aims to transform people’s fundamental way of thinking through mechanisms that activate specific abilities, especially futurability, which refers to the ability to experience positive affect from decisions prioritizing the well-being of future generations over short-term gains. Futurability plays a pivotal role in shaping how present decisions are evaluated. In other words, it represents a concept that combines cognitive evaluation with affective disposition. Specifically, the Imaginary Future Generation (IFG) is one of the mechanisms to activate people’s futurability by enabling individuals to mentally project themselves into future contexts [15].

The first empirical study to demonstrate that IFGs could be utilized to improve intergenerational equity was conducted by Kamijo et al. [7], who designed an intergenerational sustainability dilemma game (ISDG) in their study. It is an incentive-compatible game. Three respondents were allocated to each group. Each group represented a different generation. Each group was given ten minutes to discuss its choice between Option A (a selfish option) and Option B (a sustainable option). Their study showed that 28% of the groups without a representative of the future generations chose Option B, whereas 60% of the groups with a representative of future generations chose Option B. It was found that playing the roles of representatives of future generations could induce futurability, and it not only affected the individual’s decision-making for future generations but also other respondents within the same generation. Furthermore, through the deliberative analysis, Timilsina et al. [8] indicated that the existence of IFG subjects in a generation enhances the quality and quantity of deliberation, as they discuss more about reactions to earlier generations and reasons (not) to consider future generations. The study concretely identifies the concept “risk of unsucceded goodwill” as the hindering factor for choosing the sustainable option.

In order to assess the effect of IFGs on individual decision-making under various conditions, Shafen et al. [19] transformed IFG into the future ahead and back (FAB) mechanism, in which participants are first asked to take the perspective of the next generation and articulate their requests to the previous generation, and then make actual decisions after going back to the original generation. The study combined one-person ISDG with FAB and adopted the conditional information lottery (CIL) method. Each participant plays 36 different situations/games, consisting of 35 fictional situations and a single true situation that affects their own payoff and others’, and can see how previous individuals chose in the sequence they belong to. Those fictional situations are designed to control for the retrospective factor (e.g., the percentage of previous generations choosing unsustainable options) and the prospective factor (e.g., how many subsequent generations can still receive a positive payoff if everyone continues to choose the unsustainable option). They found that FAB subjects are 15.8% more likely to choose the sustainable option than those in the basic ISDG. Additionally, it suggests that such innovative institutional designs may be essential for addressing pressing threats to intergenerational sustainability, as conventional policy tools alone may be insufficient to prevent long-term unsustainable outcomes. Notably, FAB and IFG interventions to activate futurability are essentially both temporal perspective-taking.

Except for ISDG experiments, IFGs have been widely applied in workshop-based studies focusing on sustainability. Early research demonstrated that IFGs redirected attention from short-term concerns to long-term environmental issues, such as water pollution [20], alleviated nearsightedness in urban planning [21], and promoted policy choices beneficial to future generations [22,23]. Deliberative field experiments further demonstrated that adopting the perspective of future generations increases the likelihood of selecting sustainable options in waste management [24]. In some studies, an intergenerational retrospective viewpoint (IRV) was also adopted, as seen in forest management [25] and financial policy [26]. The IRV approach operates by having individuals first evaluate unrelated historical decisions to evoke feelings of gratitude or regret toward past generations. Subsequently, participants are asked to adopt the perspective of a future generation to assess and choose current policy options, which is IFG.

Some studies tried to examine the role of personality on the effect of IFGs. They examined the social value orientation (SVO), where individuals make a series of choices on how to allocate resources between themselves and another person, suggesting that people with a pro-social personality have a longer time perspective and are more likely to choose intergenerationally sustainable options [21,27]. These studies highlighted that stakeholder participation and future-oriented perspectives influence individual thinking and strengthen motivation for long-term policy commitments.

More recent studies have extended this evidence across diverse policy domains. In municipal water systems, IFGs produced markedly different visions for 2050 and stimulated crisis awareness, enabling more flexible long-term policy formulation [28]. A large-scale survey found that IFG perspectives increased acceptance of higher water rates, especially among respondents with a pessimistic future outlook [29]. IFGs have also been applied to decarbonization, where workshops combining IFGs with systems thinking encouraged participants to propose new institutional mechanisms [30]. In food systems, by taking the perspectives of people from past, current, and future viewpoints, FD induced people to make persistent changes in sustainable food consumption [31] and shifted people’s preferences toward transformative policy changes by forming future-oriented group identity [32]. Furthermore, imagining oneself as a resident in 2050 enhanced SDG engagement, cooperation, and futurability [33]. Together, these findings suggest that IFGs help overcome present-oriented shortsightedness, foster long-term thinking, and promote sustainable decision-making. This implies that FD offers a possible pathway for socioeconomic challenges by explicitly incorporating future-oriented perspectives.

Despite these positive findings, the effectiveness of IFG interventions appears to be context-dependent [15]. For example, a study by Shahrier et al. [27] in Bangladesh found that while IFGs had a significant impact on residents in rural areas (less capitalistic), they had little effect on their urban counterparts (more capitalistic). This suggests that cultural or socioeconomic factors may mediate the effectiveness of IFG interventions. In another case, IFG interventions were not found to be effective in increasing participants’ willingness to donate to environmental organizations, regardless of whether the NGO focused on short-term (e.g., air pollution) or long-term (e.g., climate change mitigation) improvements [34]. Nevertheless, when viewed collectively, these findings indicate that IFGs help overcome present-oriented shortsightedness. More importantly, it suggests that FD offers a viable pathway for addressing systemic socioeconomic challenges by explicitly incorporating future-oriented perspectives.

### 2.3 Behavioral interventions for sustainability

Beyond the Imaginary Future Generation (IFG) mechanism, there are other behavioral interventions that have been explored to promote sustainable actions. Thaler and Sunstein [9] argue that a key strategy for addressing climate change must rely on incentives and information disclosure. This approach aims to change how consumers receive feedback, allowing the public to clearly perceive the potential costs of pollution. For instance, creating a greenhouse gas database, driven by social pressure from environmental groups, the media, and the public, can motivate companies to reduce emissions at a low cost to protect their brand image.

Building on this, a review paper [5] highlights that a growing number of studies leverage social norms to promote pro-environmental behaviors. They contend that a fundamental challenge of environmental issues is their temporal lag; thus, by encouraging people to engage in “future thinking,” the psychological distance to these problems is reduced, which in turn increases their concern and willingness to act pro-environmentally. Furthermore, Milfont and Schultz [5] found that social norms significantly influence environmental decision-making. As Timilsina et al. [8] note, the IFG creates a dual social pressure: it introduces a new, positive social norm to conform to sustainability goals and mitigates the existing group norm of prioritizing short-term self-interest.

The Theory of Planned Behavior (TPB) is widely applied as a foundational framework for examining individuals’ behavior. According to Ajzen [35], the TPB posits that behavior is primarily driven by an individual’s intention to perform it, which is in turn influenced by three factors: attitudes toward the behavior, perceived subjective norms, and perceived behavioral control. Building on this framework, recent research has extended TPB to investigate how individuals form intentions regarding climate change mitigation actions. Shaw et al. [34] found that attitude, subjective norms, perceived behavioral control, and futurability were key factors that significantly influence environmental donation intentions. Notably, Ghafouri [36] was the first study to specifically apply TPB to the public’s support for carbon tax policy, emphasizing attitudes and perceived control as the predictors for support. They also reveal the public preference for the carbon tax scheme, including the use of carbon tax revenues, demonstrating the model’s relevance for understanding public responses to climate policy instruments.

## 3  Materials and methods

There are a few interventions to activate futurability under FD, including IFG, FAB, IRV, or deliberative institutional roles. There are only a few studies of FAB [19] or IRV [25,26], whereas more IFG research has shown evidence across diverse domains. This study employed a survey experiment to quantify the effectiveness of IFG on the preference for carbon tax schemes because of the following reasons. First, the mechanism of both IFG and FAB is taking the future-generation perspective. FAB was adopted in Shahen et al. [19] because the participants had to play 36 rounds of one-person ISDG, which consisted of various sequences of generations. However, climate change is a contemporary issue, and the carbon tax policy in Taiwan has not yet been launched, so it does not involve prior generations’ choices. Second, the selection of historical examples for retrospective framing is an important factor affecting outcomes [15], but we are unsure how to find appropriate examples in Taiwan. Third, this study aims to investigate the public preference for carbon tax schemes, distinct from deliberation workshops (e.g., deliberative institutional roles), which primarily focus on shaping policies or the IFG mechanism, using a quantitative analysis. Thus, the IFG is most appropriate for this online survey-experiment study. Specifically, IFG/control respondents can select paired comparisons among the five schemes. For the IFG group, we used five open-ended questions (narrative imagination) guiding their imagination about future generations, and asked them to assume the role of future generations in 2025 (role enactment).

### 3.1 Research design

To test our hypotheses, we designed an online survey experiment via the Qualtrics platform and sent it out through the Web Survey NTU platform maintained by National Taiwan University. Initially, the questionnaire was tested by a small group of respondents, a focus group, and a further group of 100 respondents. The final survey experiment was completed on December 9, 2021, and we collected a total of 950 valid responses by checking repeated IP addresses. While we aimed to secure a diverse pool of respondents, a probability sampling strategy was not feasible given the nature of the online survey platform. Instead, we employed a quota sampling strategy to ensure sample diversity and mitigate the self-selection bias often associated with online opt-in panels.

Based on the membership pool of the Web Survey NTU, our sampling frame was stratified to match the demographic characteristics of the general population in Taiwan. Specifically, we set quotas for gender, age, and residential area based on the government census data released in 2020. This approach allowed us to obtain a sample that structurally resembles the population, balancing the trade-off between logistical feasibility and representativeness. It is important to note that our primary analytical focus is on estimating the average treatment effect (ATE) of the IFG rather than generating precise point estimates for public opinion. Recent methodological literature suggests that while convenience or quota samples may yield similar experimental treatment effects [37].

An NT$50 voucher, redeemable at a convenience store, was offered as an incentive for completing the questionnaire using the matching code provided. In some previous IFGs studies, the respondents were given gains depending on their choices [7]. However, because climate change is a long-term issue and the effects of carbon tax policies unfold over time, it is difficult to determine an appropriate monetary payment corresponding to the specific policy chosen by respondents. The environmental, economic, and social impacts of such policies are hard to quantify, and individual-level effects vary depending on characteristics such as income. Additionally, the losses caused by climate change are both substantial and uncertain. Given these challenges, and consistent with many IFG studies in which all respondents received the same payment in policy-making processes, this study provided all participants with a fixed incentive.

This research was approved by the Institutional Review Board for Humanities & Social Science Research/IRB-HS Academia Sinica (AS-IRB-HS-21057). All methods were carried out according to relevant guidelines and regulations. The authors had no access to the information that could identify participants during or after data collection. Electronic informed consent (via an online ‘I agree’ click) was obtained from all participants prior to their participation. Respondents were required to read the informed consent form displayed on the Qualtrics platform before proceeding. Only adult participants (aged 18 and above) were included in this study; therefore, parental or guardian consent was not required.

### 3.2 Questionnaire design

There are four parts to our questionnaire: Part 1 contains information and questions related to climate change and carbon emissions reduction; Part 2 presents an experiment on IFGs; Part 3 covers carbon tax schemes; and Part 4 collects respondents’ demographic and socioeconomic information, along with their climate-related attitudes and behaviors.

Part 1 cites the IPCC Report [38] to explain the causes and impacts of climate change, and describes Taiwan’s carbon reduction targets and the current policies in place to achieve these targets. Greenhouse gas concentrations are broadly similar worldwide, yet climate change impacts vary across regions. To address information asymmetry—such as differing perceptions of climate change—this section ensures participants clearly understand that current climate change is primarily driven by human activities and outlines Taiwan’s relevant legal provisions. The following is part of the information:

“According to the Fifth Assessment Report of the Intergovernmental Panel on Climate Change (IPCC), ‘Human activities are the main cause of warming observed over the past 50 years.’…In 2015, the Taiwanese government enacted the ‘Greenhouse Gas Reduction and Management Act.’ Article 4 specifies that long-term greenhouse gas reduction targets aim to reduce emissions in 2050 to below 50% of the 2005 level…”

In fact, at the time of the study, the Greenhouse Gas Reduction and Management Act was the primary legal framework, which was subsequently amended and renamed as the Climate Change Response Act in 2023. After reading, participants were asked to answer related questions. Please refer to S2 Text for the complete questionnaire.

In our questionnaire, Part 2 is the core element as it requests respondents to exercise a task of IFGs and then ask their preference for different schemes of carbon tax over the non-carbon alternative in Part 3. For this purpose, the respondents are randomly assigned to either a control group or an experimental group (i.e., the IFGs group). Those who were assigned to the experimental group were instructed to engage in the following exercise: “Please imagine, for a minute, that you can use a time machine that will transport you to Taiwan in 2050. You will experience life and the environment in 2050, meet the inhabitants in 2050, and feel the changes in the environment.” To ensure that the respondents did conduct the exercise of imagining the future, they were asked to write down what the environment in 2050 would be like, including the scenery, changes in the environment, air quality, biological environment, life for the people there, and so on. It’s required to respond to those questions (they cannot proceed to the next part if they do not write anything), although we did not evaluate their responses individually. The average treatment effect (ATE) of the IFG was evaluated later. Upon completing the questions related to IFGs, respondents were told: “Now, please take the time machine and travel back to the present. You are now a representative for future generations. The carbon emissions behavior of the current generation affects climate change in the future. Next, please compare the following carbon reduction policies.” They then proceeded to Part 3, which presented various carbon tax policy options.

The respondents not selected for the IFGs experiment were assigned to the control group. They skipped the exercise of IFGs and IFGs-related questions and directly proceeded to Part 3 of the questionnaire. Part 3 of the questionnaire is the choice of no/carbon tax schemes. As shown in Table 1, we designed four hypothetical carbon tax schemes and a no-carbon-tax scheme as a comparator. According to Shaw et al. [39], to address the potential adverse effects of carbon taxes on economic growth and income distribution (that is, to improve fairness), four carbon tax schemes (Schemes A to D) were established based on the principle of revenue neutrality. These schemes are differentiated by their revenue recycling mechanisms, which aim to achieve different policy objectives: (1) **Scheme A (Tax-reduction oriented),** which focuses on economic efficiency by reducing existing distortionary taxes, including VAT, income tax, and social security contributions paid by employers; (2) **Scheme B (Mixed approach),** combining tax reduction with uniform lump-sum rebates to balance both economic growth and social equity; (3) **Scheme C (Redistribution oriented),** prioritizing social fairness through uniform lump-sum rebates, specifically aimed at offsetting the regressive nature of carbon taxes; (4) **Scheme D (Baseline/No-recycling)**, serving as a fiscal baseline where tax revenue enters the national treasury to reduce the deficit.

**Table 1:** The attributes and impacts of carbon tax and non-carbon tax schemes.

Impact	Scheme A	Scheme B	Scheme C	Scheme D	Scheme E
	Carbon Tax	Carbon Tax	Carbon Tax	Carbon Tax	No Carbon Tax
Tax revenue	The tax revenue will be used to reduce the VAT rates, household income tax rates, and social security contributions	The tax revenue will be used to reduce the VAT rates and provide lump-sum rebates of NT$80,000 per person	The tax revenue will be used for lump-sum rebates of NT$100,000 per person	All tax revenue will go to the treasury.	No change
Carbon emissions compared to 2005	Reduced by 50%	Reduced by 50%	Reduced by 50%	Reduced by 50%	Increased by 41%
The annual income of the lowest-income group	Increased by 4.2%	Increased by 7.5%	Increased by 5.9%	Increased by 2.9%	No change
GDP	Increased by 4.1%	Increased by 4.6%	Increased by 3.4%	Reduced by 1.9%	Reduced by 0.1%
Electricity bills per year per person	Increased by NT$11,000	Increased by NT$11,000	Increased by NT$11,000	Increased by NT$11,000	No change

In the no-carbon-tax scenario (Scheme E), we assumed the continuation of the Feed-in-Tariff (FIT) policy. This instrument has served as Taiwan’s primary policy tool for supporting renewable energy, through price subsidies, since the enactment of the Renewable Energy Development Act in 2009. Taiwan’s formal commitment to emission reduction was solidified by the Greenhouse Gas Reduction and Management Act in 2015, which established a long-term goal to reduce emissions to at least 50% below 2005 levels by 2050. However, its effectiveness has been limited because electricity prices in Taiwan often fail to reflect the actual costs of renewable generation [39].

The effects of each scheme were simulated using the E3ME (Energy-Environment-Economy Global Macro-economic) model [39]. Unlike traditional CGE models, E3ME is a demand-driven global macro-econometric model that captures the dynamic interactions between energy, environment, and the economy, making it particularly suitable for simulating the long-term socio-economic impacts of achieving Taiwan’s 2050 targets. As shown in Table 1, the comparison table presented four distinct attributes: (1) **Environmental impact**, showing whether carbon emissions would increase or decrease after the implementation of the carbon tax schemes; (2) **Social impact**, indicating whether the annual income of the lowest 20% income group would increase or decrease; (3) **Economic impact**, chowing whether the domestic GDP would increase or decrease in 2050; and (4) **Individuals economic impact**, detailing whether the electricity bills per year per person would increase or decrease in 2050. To prevent attribute order bias, we created two versions of the table with the order of the social and economic attributes reversed. The survey platform randomly assigned each respondent to one of the two table versions and randomly arranged the schemes on the left or right side of the screen to prevent display bias. Given that the survey was conducted online and primarily on mobile devices, the questionnaire was optimized to fit standard mobile screen widths.

Before making a choice, respondents were presented with the following statement: “In 2020, a carbon tax was levied at NT$420 per ton of carbon dioxide, equivalent to an increase of NT$0.21 per kWh of electricity and an increase of NT$0.84 per liter of petrol.” Participants were then asked to choose between two schemes across all ten possible unique pairs of the five schemes (S1 Text shows an example of the carbon tax comparison table as displayed on a mobile phone). The core focus of this study was on respondents’ choices between a carbon tax scheme (Schemes A to D) and the no-carbon-tax scheme (Scheme E). We expect that respondents would prefer a carbon tax scheme if they were assigned to the experimental group and nudged by the IFG exercise.

## 4  Results and discussion

Table 2 presents descriptive statistics for all variables across the control and IFG groups. The sample comprises 950 participants, including 481 in the control group and 469 in the IFG group. Most participants were aged 30–40, and females accounted for 53.16% of the sample. Most participants were aged 30–40, and females accounted for 53.16% of the sample in both groups. The majority held a university degree (59.16%), while those with a junior high school education or below were least represented (3.58%). Monthly income was most commonly between NT$20,000 and NT$40,000 (36.84%). Students and homemakers accounted for 8.63% and 10.74% of participants, respectively. Few participants live in the hills (5.47%) or by the sea (16.21%), and 62% reported having no cohabiting children.

**Table 2:** Descriptive statistics for all variables across the control and IFG groups.

Variables		The control group		The IFG group	
		N = 481		N = 469	
		N	%	N	%
Age	18 ~ 19	1	0.11	1	0.11
	20 ~ 29	103	10.84	108	11.37
	30 ~ 39	175	18.42	164	17.26
	40 ~ 49	134	14.11	140	14.74
	50 ~ 59	23	2.42	25	2.63
	60 ~ 69	34	3.58	24	2.53
	70 ~ 79	11	1.16	7	0.74
Monthly personal income (NT$10,000)	1	106	11.16	90	9.47
	3	171	18.00	179	18.84
	5	119	12.53	132	13.89
	7	54	5.68	45	4.74
	9	15	1.58	13	1.37
	11	7	0.74	4	0.42
	13	4	0.42	2	0.21
	15	1	0.11	1	0.11
	19	1	0.11	2	0.21
	21	3	0.32	1	0.11
Gender	Female	245	25.79	260	27.37
	Male	236	24.84	209	22.00
Living in the hills	No	454	47.79	444	46.74
	Yes	27	2.84	25	2.63
Living by the sea	No	413	43.47	383	40.32
	Yes	68	7.16	86	9.05
The order of the scheme attributes	The lowest income group at the top	248	26.11	224	23.58
	Electricity bills at the top	233	24.53	245	25.79
Number of cohabiting children	0	290	30.53	299	31.47
	1	75	7.89	57	6.00
	2	91	9.58	87	9.16
	3	22	2.32	23	2.42
	4	1	0.11	2	0.21
	5	2	0.21	1	0.11
Education	Junior high school and below	21	2.21	13	1.37
	Senior high or 3-year occupational school	46	4.84	45	4.74
	5-year occupational college	45	4.74	56	5.89
	University	272	28.63	290	30.53
	Graduate school or above	97	10.21	65	6.84
Occupation	Student	50	5.26	32	3.37
	Homemaker	54	5.68	48	5.05
	Others	377	78.38	389	82.94
Walking or cycling instead of driving or scooter-riding	Seldom	176	18.53	175	18.42
	Often	208	21.89	204	21.47
	Always	97	10.21	90	9.47
Air pollution around my place of residence in the future will worsen	Disagree	74	7.79	60	6.32
	Agree	296	31.16	314	33.05
	Strongly agree	111	11.68	95	10.00
If I do nothing to reduce carbon, I will be condemned by others	Disagree	150	15.79	137	14.42
	Agree	269	28.32	256	26.95
	Strongly agree	62	6.53	76	8.00
The government will effectively utilize the tax revenue	Strongly disagree	93	9.79	86	9.05
	Disagree	200	21.05	208	21.89
	Agree	156	16.42	150	15.79
	Strongly agree	32	3.37	25	2.63
Has there been any discussion about responses to carbon taxes at your workplace**?**	No discussion	272	28.63	249	26.21
	Yes, but unclear	155	16.32	174	18.32
	Yes, plans are being put in place	54	5.68	46	4.84
Has there been any impact of climate change on your industry?	Unclear	113	11.89	112	11.79
	No impact	221	23.26	190	20.00
	Negative impacts	68	7.16	83	8.74
	Positive impacts	79	8.32	84	8.84

In both groups, similar proportions of participants report frequently walking or cycling instead of using cars or scooters (21.89% in the modern group and 21.47% in the IFG group), while about 18% in each group seldom do so. Overall, 64.21% of participants agree that air pollution around their residence will worsen in the future, whereas only 14.11% disagree. Over half (55.26%) of the total participants agree that if they do nothing to reduce carbon emissions, they will be condemned by others, compared with 30.21% who disagree. Fewer than half (42.95%) disagree that the government will effectively utilize the tax revenue, and only 6% strongly agree with this statement. A majority (54.84%) report that there is no discussion of the carbon tax at their workplace, while only 10.53% indicate that discussions and plans are in place. Regarding industry impacts, 43.26% perceive no effect of climate change, whereas similar shares report negative (15.89%) and positive (17.16%) impacts.

### 4.1 The preferences for carbon tax schemes

**Fig 1:**
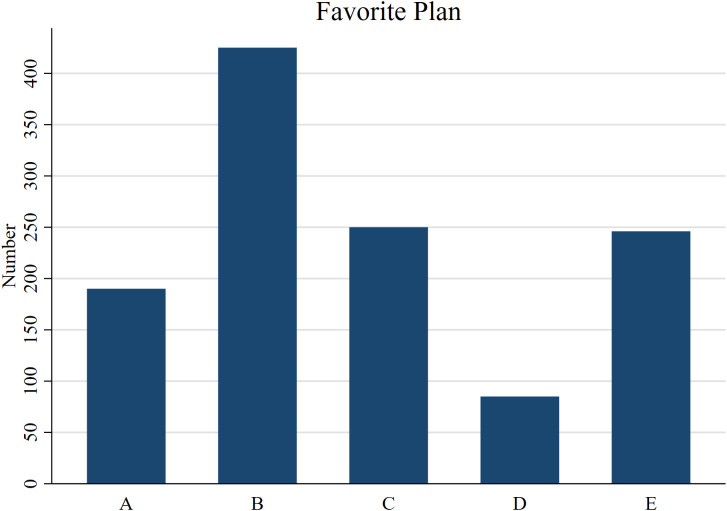
Statistical chart of the respondents’ favorite carbon tax scheme.

Fig 1 shows the carbon tax schemes preferred by the respondents in this research. Each participant made paired comparisons among the five schemes (A to E), choosing one scheme from each pair. The selected scheme in each pair received one point. After ten rounds of paired comparisons, the scheme with the highest total score was identified for each participant. However, ties may occur in the selection since the five schemes are mutually independent. In total, there were 950 observations with a clear preference plus 246 observations with ties, resulting in a total of 1,196 observations.

The results showed that Scheme B was chosen as the most preferred carbon tax scheme by 35% (425 out of 1,196 total observations) of respondents. Conversely, Scheme D was the least favored, being the top choice for only 7% (85 out of 1,196 total observations) of respondents. The tax revenue of Scheme B would be used to reduce the VAT rates and provide lump-sum rebates of NT$80,000 per person. Scheme C was the second most popular choice, and its tax revenue would be used to provide lump-sum rebates of NT$100,000 per person. Although the lump-sum rebate of Scheme B is less than that of Scheme C, the social and economic impacts of Scheme B are better than those of Scheme C.

### 4.2 Comparison of paired options between a carbon tax scheme and a non-carbon tax scheme

**Fig 2:**
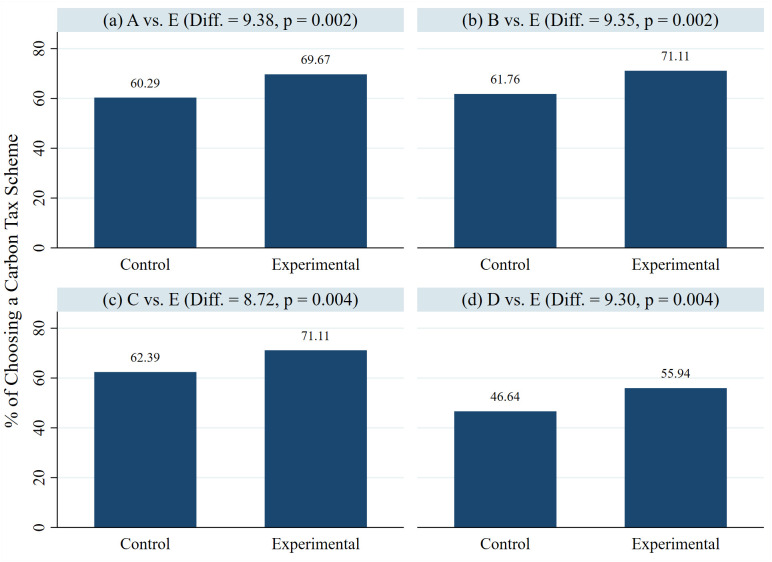
Respondent preferences for carbon tax schemes over no tax: Control vs. experiment group proportions. (a) to (d) also shows the p-value of two-tailed t-tests, respectively.

The dataset of this study is in the S1 File. As shown in Fig 2, respondents in the IFGs group were more likely to choose either Scheme A, B, C, or D against Scheme E compared to those in the control group. More importantly, the differences between the two groups are all statistically significant at p < 0.01 level across 4 comparisons with Scheme E as the baseline. In other words, the respondents in the IFGs group have become more favorable to a carbon tax scheme that is better for the environment. This result confirms our hypothesis.

Most respondents in the control group even prefer Scheme E to Scheme D. It is noteworthy that both the control and treatment groups of respondents in Fig 2d show lower support for Scheme D compared to their support for the other three carbon tax schemes. One major difference between Scheme D and other carbon tax schemes is how the tax revenue will be used. In Schemes A to C, the tax will be used to either reduce the tax rates or to rebate to individuals, while in Scheme D, it will go to the treasury. In other words, respondents would be less supportive if the tax revenue is levied by the government but not allocated to other purposes.

In this study, the Theory of Planned Behavior (TPB) was adopted as a framework to examine individuals’ pro-environmental behavior intentions, which are constructed by attitudes, subjective norms, and perceived behavioral control, in support of the carbon tax schemes. Attitudes towards climate change mitigation are assessed by whether respondents agree that air pollution in their residential area will worsen in the future, and their mitigation behavior, such as frequently walking or cycling instead of driving or riding a scooter. Subjective norms, which refer to the perceived expectations from others if an individual performs a particular behavior, are measured by whether respondents agree that they would be criticized by others if they did nothing to save energy or reduce carbon emissions. Perceived behavioral control refers to an individual’s perceived ability to carry out a behavior. Moreover, when carbon taxes are implemented, businesses may face increased costs and additional administrative burdens. Thus, whether a respondent has discussed carbon tax responses at work may reflect their perception of how much control they or their organization has in handling such policies.

In terms of the social and economic variables, this research controls not only for standard factors such as gender, age, education, and income but also includes “number of cohabiting children” as well as “student” and “homemaker” as separate occupational categories. Since the goal of this study is to assess the effect of IFGs, cohabiting children are the closest future generation; participants living with children may naturally be more concerned about intergenerational issues. One of the reasons why “student” and “homemaker” are considered distinct occupations is that respondents in these occupations have no income. Consequently, income-related effects may not be directly observable in their decision-making, warranting their separation from other occupational groups. In addition, we control for geographic variables related to vulnerability to climate change. Given that carbon taxes aim to mitigate climate risks, individuals living near the coast or in hilly areas may be more likely to support carbon tax policies.

Table 3 presents the logit estimates of the marginal effects for paired options, specifically comparing carbon tax schemes (A, B, C, or D) with a non-carbon tax scheme (E). The marginal effects refer to the probability of choosing a carbon tax scheme. The results of logistic regression with socioeconomic and other control variables further confirmed our hypothesis, that is, respondents experiencing IFGs would be more supportive of the carbon tax plan than those without such an experience (see S1 Table). The results for the first variable, IFGs, indicate that IFGs have a positive impact after the social and economic variables are controlled. The marginal effects are all greater than 0.11, which means that respondents who are introduced to IFGs are 11% more likely to choose Scheme A, B, C, and D compared to those who are not introduced to IFGs. In other words, the introduction of IFGs can increase a respondent’s acceptance of the carbon tax scheme.

**Table 3:** Marginal effects for choosing Schemes A to D against Scheme E.

Variables	Marginal effect estimates (SE)			
	Model 1 (A vs. E)	Model 2 (B vs. E)	Model 3 (C vs. E)	Model 4 (D vs. E)
IFGs	0.124*** (0.030)	0.112*** (0.030)	0.110*** (0.030)	0.126*** (0.031)
Age	0.003* (0.002)	0.004** (0.002)	0.002 (0.002)	0.003* (0.002)
Income^a^	0.004 (0.007)	−0.005 (0.006)	−0.000 (0.007)	−0.003 (0.006)
Male^b^	−0.013 (0.033)	−0.004 (0.033)	0.001 (0.033)	0.049 (0.035)
Living in the Hills^b^	0.040 (0.069)	0.075 (0.069)	−0.030 (0.066)	0.033 (0.073)
Living by the Sea^b^	−0.045 (0.041)	−0.027 (0.041)	−0.032 (0.041)	−0.010 (0.045)
The Order of the Scheme Attributes^c^	0.071** (0.030)	0.068** (0.030)	0.048 (0.030)	0.023 (0.032)
Number of Cohabiting Children	−0.014 (0.018)	−0.008 (0.018)	−0.001 (0.018)	0.003 (0.019)
College^b^	0.002 (0.042)	0.040 (0.041)	0.068* (0.041)	0.028 (0.044)
Occupation^b^				
Others	Ref	Ref	Ref	Ref
Student	0.073 (0.064)	0.140** (0.067)	0.089 (0.065)	0.011 (0.065)
Homemaker	−0.072 (0.056)	−0.082 (0.055)	−0.002 (0.058)	−0.031 (0.060)
N	950			

* p < 0.1, ** p < 0.05, *** p < 0.01.

a Income: NT$10,000 per unit.

b Male, living in the hills, living by the sea, college, and occupation are dummy variables.

c The order of the scheme attributes, from top to bottom, is carbon emissions, electricity bills per year per person, and GDP, with the income of the lowest income group at the bottom, coded as 1. The alternative order places carbon emissions at the top, followed by the income of the lowest income group and GDP, with electricity bills per year per person at the bottom, coded as 0.

As for the social and economic variables, age, education, and occupation were positive and statistically significant in some situations. The older the respondents are, the more likely they will choose Scheme A, B, and D. Regarding education, the marginal effect of having a college degree is significantly positive only when respondents choose between Schemes C and E. Specifically, individuals who graduated from college are 6.8% more likely to choose Scheme C. Scheme B is preferred more by respondents whose occupation is “student” than by those in other occupational groups. Regarding the order of the carbon tax scheme attributes, the marginal effects are significantly positive when respondents choose between Schemes A and E, and between Schemes B and E. When the attribute of “Electricity bills per year per person” is followed by “GDP” and “Income of the lowest income group”, the respondents are 6.8% more likely to choose carbon tax schemes (A and B). This is probably because the respondents might focus on the attribute close (at the bottom) to the selection button, or consider the attribute “Income of the lowest income group” to be more important, and its position (at the bottom) reminds them.

### 4.3 Comparison of the key variables between the control and IFGs group

To investigate and compare the key variables influencing respondents’ decisions between the control group and the IFGs group, an ordered logit model was applied separately to each group. The analysis assessed the respondents’ frequencies of selecting Scheme E (the non-carbon tax scheme) over the other four schemes. Thus, the outcome variable will range from 0 to 4. The results in Table 4 are presented as an odds ratio (see S2 Table for the complete marginal effect results). An odds ratio less than 1 (greater than 1) indicates that as the independent variable increases, respondents are less (more) likely to select Scheme E. This implies a higher likelihood of choosing a carbon tax scheme (or the non-carbon tax option, respectively).

**Table 4:** Odds ratios of the ordered logit model for the control and IFGs group.

Variables	Odds ratios (SE)			
	Control Group		IFGs Group	
Age	0.996	(0.010)	0.975**	(0.011)
Income^a^	1.067*	(0.036)	0.924**	(0.036)
Male^b^	0.824	(0.151)	1.024	(0.192)
Living in the Hills^b^	0.861	(0.333)	0.837	(0.275)
Living by the Sea^b^	0.818	(0.198)	1.502*	(0.312)
The Order of the Scheme Attributes^c^	0.734*	(0.122)	0.868	(0.155)
Number of Cohabiting Children	1.048	(0.108)	0.996	(0.111)
College^b^	0.924	(0.231)	0.713	(0.173)
Occupation^b^				
Others	Ref	Ref	Ref	Ref
Student	0.878	(0.284)	0.670	(0.228)
Homemaker	1.252	(0.426)	1.106	(0.434)
Walk^d^	0.855	(0.105)	1.108	(0.146)
Air Pollution^d^	0.720**	(0.100)	0.961	(0.154)
Subjective Norms^d^	0.892	(0.140)	0.688**	(0.107)
Government Efficiency^d^	0.929	(0.103)	1.027	(0.119)
Behavioral Control^d^	1.251	(0.171)	1.522***	(0.216)
Industry Vulnerability^b,d^				
No impact/Unclear	Ref	Ref	Ref	Ref
Positive Impacts	0.734	(0.187)	0.793	(0.207)
Negative Impacts	0.824	(0.237)	0.948	(0.208)
N	481		469	

* p < 0.1, ** p < 0.05, *** p < 0.01

a Income: NT$10,000 per unit.

b Male, living in the hills, living by the sea, college, occupation, and industry vulnerability are dummy variables.

c The order of the scheme attributes, from top to bottom, is carbon emissions, electricity bills per year per person, and GDP, with the income of the lowest income group at the bottom, coded as 1. The alternative order places carbon emissions at the top, followed by the income of the lowest income group and GDP, with electricity bills per year per person at the bottom, coded as 0.

d The label sheet of the S1 File reports the operationalizations of variables.

As Table 4 demonstrates, the older an IFG respondent is, the fewer the number of times he or she will select Scheme E, *i.e.,* the more that he or she will prefer a carbon tax scheme, whereas age is insignificant for the control group respondents. The higher the income of the control group respondents, the more times they will select Scheme E. The process of IFGs causes a reverse effect, *i.e.,* the higher the income of the IFGs group respondents, the fewer times they will select Scheme E. That is to say, the IFGs process will persuade the high-income and older respondents in the IFGs group to select a carbon tax scheme. Besides, Scheme E will be selected a greater number of times by the IFGs respondents who live by the sea. The reason appears to stem from the characteristics or livelihoods of the residents, rather than concerns about the impacts of climate change.

In the control group, those who agree more that “the air pollution around their place of residence will worsen in the future” would select Scheme E fewer times. That means that without IFGs, people who are more concerned with the air quality will prefer a carbon tax. In addition, after experiencing IFGs, the respondents who agree more that “if they do nothing to reduce carbon emission will be condemned by others” would select Scheme E fewer times, which means they prefer a carbon tax.

**Fig 3:**
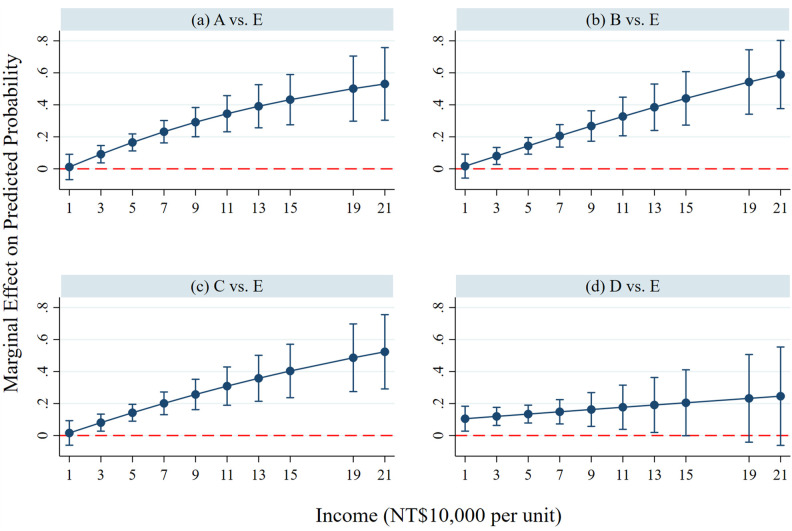
The marginal effect of IFGs on support for the carbon tax scheme across Income differentials.

According to the results in Table 4, the income of the respondents can increase and decrease the number of times they choose non-carbon tax schemes in the control and the IFGs groups, respectively. To further examine whether the process of IFGs has heterogeneous effects across different income groups, we interact IFGs with income in Table 5. Then, we draw the marginal effect plots to illustrate how the marginal effect of IFGs varies across respondents’ differences in income for four paired options (AE, BE, CE, and DE). Fig 3a-3c show that when respondents’ income increases, the marginal effect of IFGs on their support for the carbon tax scheme (i.e., A, B, and C) becomes more positive and statistically significant at a 90% confidence level. Individuals who have higher incomes would be more likely to select the carbon tax scheme after experiencing the IFGs process, as we can also observe in S2 Table.

**Table 5:** Estimates of the interaction between IFGs and income for choosing Schemes A to D against Scheme E.

	AE	BE	CE	DE
IFGs	−0.129	−0.071	−0.077	0.399*
	(0.253)	(0.255)	(0.254)	(0.230)
Income^a^	−0.039	−0.077**	−0.050	−0.026
	(0.034)	(0.033)	(0.034)	(0.033)
IFGs × Income	0.180^***^	0.149***	0.149***	0.029
	(0.055)	(0.053)	(0.054)	(0.047)
Age	0.015^*^	0.017**	0.009	0.014*
	(0.008)	(0.008)	(0.008)	(0.008)
Male^b^	−0.082	−0.033	−0.013	0.198
	(0.153)	(0.156)	(0.153)	(0.143)
Living in the Hills^b^	0.209	0.380	−0.119	0.142
	(0.314)	(0.327)	(0.311)	(0.300)
Living by the Sea^b^	−0.191	−0.109	−0.134	−0.038
	(0.189)	(0.194)	(0.194)	(0.186)
The Order of the Scheme Attributes^c^	0.339^**^	0.331**	0.233	0.095
	(0.141)	(0.143)	(0.142)	(0.133)
Number of Cohabiting Children	−0.074	−0.049	−0.014	0.010
	(0.082)	(0.084)	(0.083)	(0.077)
College^b^	0.001	0.181	0.310	0.113
	(0.190)	(0.195)	(0.190)	(0.179)
Occupation^b^				
Others	Ref	Ref	Ref	Ref
Student	0.347	0.649**	0.417	0.040
	(0.286)	(0.311)	(0.296)	(0.267)
Homemaker	−0.283	−0.358	0.023	−0.125
	(0.253)	(0.257)	(0.268)	(0.244)
N	950			

* p < 0.1, ** p < 0.05, *** p < 0.01

a Income: NT$10,000 per unit.

b Male, living in the hills, living by the sea, college, occupation, and industry vulnerability are dummy variables.

c The order of the scheme attributes, from top to bottom, is carbon emissions, electricity bills per year per person, and GDP, with the income of the lowest income group at the bottom, coded as 1. The alternative order places carbon emissions at the top, followed by the income of the lowest income group and GDP, with electricity bills per year per person at the bottom, coded as 0.

### 4.4 Discussion

This research investigates whether introducing IFGs will increase the probability of the respondents choosing carbon tax schemes. A logit model was used to analyze the data regarding the choices that the respondents made when one of the carbon tax schemes, that is, Scheme A, Scheme B, Scheme C, or Scheme D, was paired with the non-carbon tax scheme, i.e., Scheme E. Moreover, an ordered logit model was used for the experimental (IFGs) group and the control group (without IFGs) to analyze the impact of the key variables on the number of times Scheme E (non-carbon taxes) was selected by the respondents.

As we expect, the introduction of IFGs can increase a respondent’s acceptance of the carbon tax scheme by 11%. Our finding resonates with previous work, which has suggested that people will have fewer or delayed gains after imagining the future [7,40], and with prior studies that effectively induce “futurability” that helps long-term policy design [23–25,28–30,32], but differ from those of Shaw et al. [34]. In their study, a possible reason for the ineffectiveness of IFG is that while participants were asked to imagine future living conditions, they were not explicitly instructed to play the role of future generation representatives, or give suggestions to the previous generation where they were originally located, like other IFGs or FAB studies. Additionally, we find that subjective norms are a significant factor in supporting the carbon tax among IFG respondents. The result is connected to the studies of Milfont and Schultz [5], Timilsina et al. [8], and Shaw et al. [34], who found that people perceive social norms, such as the perceived pressure from future generations, to significantly influence their pro-environmental intentions or even sustainable decision-making. This result suggests the mechanism of IFG. People sense future generations and their needs, and when they take the perspective of future generations, they intend to support the policy that favors them. Social norms are one of the mechanisms that have been adopted in some nudge studies, and that usually invoke social norms among close peers by reporting descriptive information rather than taking the perspective of others, not saying future generations [13,41].

As for socio-economic factors on pro-environmental behavior, Fuchigami et al. [31] found that household income is significantly positive but has a small effect on nonorganic vegetable consumption. However, in our study, the variable of income itself generally has no effect on the intention to accept carbon tax schemes (Tables 3 and 4), but the results of the interaction term with IFGs show that the effects from IFGs become larger for richer people (Fig 3).

## 5  Conclusions

Overall, the results show that the introduction of IFGs does significantly increase the probability by 11% that a respondent chooses the carbon tax scheme over the non-carbon tax scheme. This effect could result from adopting IFGs, as the perspective of future generations was taken into account, and people perceived pressure from future generations as social norms, which reduces the perceived psychological distance to these problems. That turned out to increase their concern and willingness to act pro-environmentally, even when their financial burden will be increased. The IFGs process will also persuade the high-income respondents to select carbon tax schemes. Therefore, the policy implications from this empirical result are that, regarding public consultation, authorities should integrate “Future Design” elements into the deliberation process. Policymakers should institutionalize exercises that prompt citizens to adopt a long-term (e.g., year 2050) perspective before soliciting their preferences on energy policies. A feasible institutional pathway is to incorporate an IFG session into public consultation procedures for carbon pricing or climate legislation. Such a module could also be embedded in citizens’ assemblies or other deliberative forums on climate policy. In addition, IFG sessions could be integrated into regulatory impact assessments or stakeholder engagement guidelines for carbon tax design. At the local level, governments could organize climate-planning workshops that include an IFG component, in which some participants are explicitly assigned the role of representing future generations. In these institutional settings, asking participants to envision a simulated future and deliberate from the standpoint of future generations may encourage more future-oriented decision-making and greater consideration of the rights and interests of those who will bear the long-term consequences of today’s policy choices, particularly with respect to carbon emissions or climate change.

A practical implementation of IFG may follow six basic steps. First, participants should be recruited to ensure broad demographic and socioeconomic representativeness. Second, they should receive a neutral pre-briefing that introduces climate change, carbon taxation, and the policy alternatives under consideration. Third, the IFG module should adopt a structured format similar to that used in this study: participants are asked to imagine themselves traveling to a future point in time, such as the year 2050, to describe the conditions they encounter, and then to deliberate; some participants may also be explicitly assigned the role of representing future generations. Fourth, this exercise may be followed by structured deliberation, moving from individual reflection to small-group discussion and then to plenary synthesis. Fifth, policy preferences should be elicited only after completion of the IFG module, so that respondents evaluate policy options from a longer-term perspective. Sixth, the process should include clear documentation and transparency regarding how participants’ views are summarized and incorporated into policy design, thereby enhancing the credibility and practical relevance of the exercise. Although such a process may require additional administrative resources and deliberative time, it can help ensure that the rights and interests of future generations are taken into account during policymaking, thereby encouraging policies that are more future-oriented.

This study demonstrates that “Scheme B,” a carbon tax bundled with a reduction in VAT rates and lump-sum rebates, is the scheme most favored among the respondents in this research. Our data demonstrate that this specific design yields the highest public acceptance, likely due to its ability to mitigate the regressive effects of taxation while providing citizens with visible economic benefits. This policy scheme also yields the best social and economic outcomes among all carbon tax schemes. According to the simulation conducted by Shaw et al. [39], the carbon tax is far more effective than FIT in terms of carbon emissions reduction in terms of climate mitigation, with models showing it could reduce carbon emissions by 50% from 2005 levels by 2050. This contrasts sharply with a no-tax scenario, which is projected to see emissions increase by 41%. While these carbon tax schemes would increase annual electricity bills by NT$11,000, it is a market-based tool that directly places a cost on emissions, unlike alternatives that a Feed-in Tariff (FIT) focuses on incentivizing renewable energy production.

In the IFGs group, the respondents’ decisions would be influenced by age and subjective norms. The more respondents agree that they would be condemned by others if they do nothing, the more respondents will prefer a carbon tax scheme. That is consistent with the literature [5]. In the control group, people who expect the air pollution around their place of residence to worsen in the future will prefer to have carbon taxes. Therefore, the IFGs experience makes most respondents, or the rich, rather than environmentalists only, feel the social pressure of climate change concerns and be willing to mitigate it. It is also discovered that the more respondents are aware of the carbon tax responses being discussed at their workplace, the less willing to accept carbon taxes. Therefore, it should be noted that the way a carbon tax is levied should be as simple as possible.

Based on our findings, we offer four concrete policy recommendations for decision-makers:

**Recommendation 1 (Consultation Design):** incorporate an IFG module into public consultations before eliciting public preferences on carbon taxes or climate policy. This recommendation is supported by our finding that the IFG treatment increased the probability of choosing a carbon tax scheme by about 11%.

**Recommendation 2 (Revenue Recycling Design):** prioritize carbon tax schemes with a visible and easy-to-understand mechanism (i.e., rebate). This recommendation is supported by the strong public support for Scheme B, which combined carbon taxation with value-added tax reduction and direct cash rebates. In implementation, policymakers should clearly communicate how revenues will be returned to citizens and make compensation mechanisms salient in policy design and public outreach. **Recommendation 3 (Simplicity and Administrative Burden):** keep policy design as simple and transparent as possible. This recommendation is motivated by our finding that respondents who discussed carbon-tax in the workplace were less likely to support carbon taxation. This correlation suggests that concerns about complexity or compliance burden may reduce acceptance. Accordingly, governments should minimize administrative complexity, explain the policy in plain language, and reduce uncertainty about compliance obligations for firms and households.

**Recommendation 4 (targeted engagement):**use IFG-based consultation strategically in outreach to groups that may be especially responsive to long-term framing. This recommendation is supported by our finding that higher-income respondents were more responsive to the IFG treatment. In practice, governments may use IFG-based consultation in stakeholder engagement with groups whose support is especially important for building durable coalitions behind carbon pricing.

The limitation of this research is that participants received a fixed payment for completing the questionnaire, regardless of which carbon tax scheme they selected, even though the schemes varied in their individual economic impact, such as increases in annual electricity bills. Since the experimental survey was adopted in this study, the mechanism of IFG cannot be further examined. Moreover, the environmental impact – carbon emission reduction – was the target of carbon tax schemes, and the economic and social impacts were simulated based on the tax rebate design, that is, the attribute levels were fixed and not varied systematically. As a result, the study could not assess how changes in specific attribute values influence individual preferences.

## Supporting information

S1 Text Example of a carbon tax comparison table displayed on a mobile phone. (DOCX)

S2 Text Questionnaire. (DOCX)

S1 File Dataset used in the analysis. (XLSX)

S1 Table Robustness analysis of the marginal effects of the Logit model for Schemes A, B, C, and D against Scheme E. (DOCX)

S2 Table Marginal effect of the ordered logit model for the control and IFGs group (the frequencies of selecting the non-carbon tax scheme E). (DOCX)
