# Supplementary material for: The impact of imaginary future generations on the preference for carbon tax schemes
Source: PLoS One. 2026 Apr 10;21(4):e0346904. doi: 10.1371/journal.pone.0346904 (PMC13068264; doi:10.1371/journal.pone.0346904)
Supplement: S1 Text — (DOCX) [file pone.0346904.s001.docx]

**Supplementary material**

**S1 Text:** Example of Carbon Tax Comparison Table Displayed on a Mobile Phone (Scheme D vs. Scheme E)


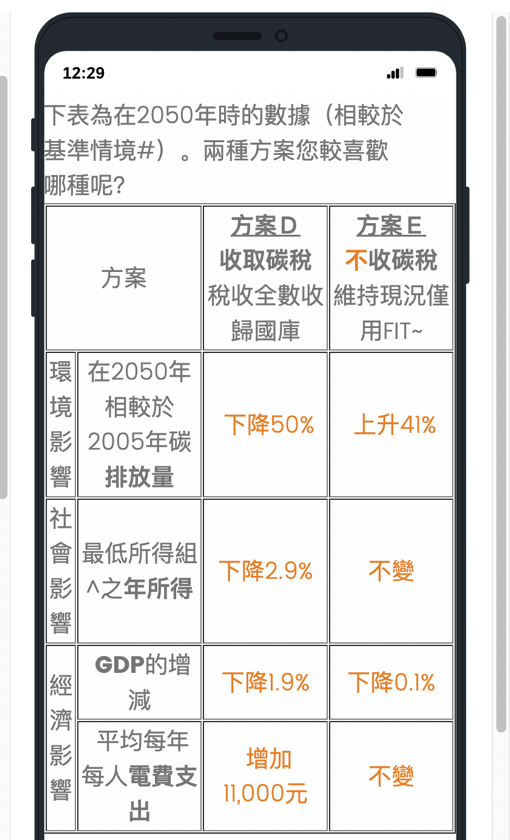

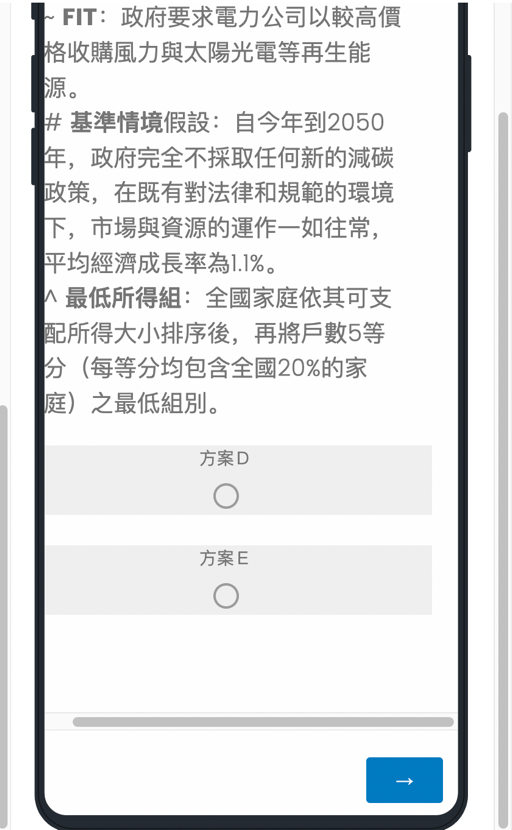


The table of environmental, social, and economic impact under two Schemes in 2050 was displayed on the participant’s mobile phone. Then ask them which one they prefer.

| Scheme | | Scheme D  Carbon Tax  All tax revenue will go to the treasury | Scheme E  No Carbon Tax  Keep using feed-in-tariff (FIT) |
| --- | --- | --- | --- |
| Environmental Impact | **Carbon emissions** compared to 2005 | Reduced by 50% | Increased by 41% |
| Social Impact | The **annual income** of the lowest-income group | Increased by 2.9% | No change |
| Economic Impact | **GDP** | Reduced by 1.9% | Reduced by 0.1% |
|  | **Electricity bills** per year per person | Increased by NT$11,000 | No change |
