# Supplementary material for: The impact of imaginary future generations on the preference for carbon tax schemes
Source: PLoS One. 2026 Apr 10;21(4):e0346904. doi: 10.1371/journal.pone.0346904 (PMC13068264; doi:10.1371/journal.pone.0346904)
Supplement: S2 Text — (DOCX) [file pone.0346904.s002.docx]

**Supplementary material**

**S2 Text:** Questionnaire

**Block: Introduction**

Hello,

We are a research team from Academia Sinica, National Cheng Kung University, Tamkang University, and National Taiwan University. We invite you to participate in a study regarding environmental change and carbon reduction. This survey takes approximately 10 minutes to complete. All responses are used solely for academic purposes. The research team strictly follows Taiwan's “Personal Data Protection Act” to ensure anonymity and protect all personal information. Participation is entirely voluntary, and you may withdraw at any time. If you have any questions after completing the survey, please feel free to contact us.

Before answering the questionnaire, please read the informed consent form for this study and indicate your agreement.

Click here to download: [**Informed Consent Form**](https://sinica.qualtrics.com/CP/File.php?F=F_0wfOzYvz4TkA7SC)

Q1.1 Do you agree to participate in this study?

- Agree
- Disagree

Q1.2 Please fill in the following information for verification of survey completion and eligibility for compensation.

Q1.3 Survey code

Q1.4 Your mobile phone number

Q1.5 Your email address

**Block: Causes and Impacts of Climate Change**

Please read the following description and answer the subsequent questions.

According to the Fifth Assessment Report of the Intergovernmental Panel on Climate Change (IPCC), “Human activities are the main cause of warming observed over the past 50 years.” Since the Industrial Revolution, humans have burned large amounts of fossil fuels such as oil and coal, increasing atmospheric CO₂ concentrations. Extensive deforestation has further reduced the number of trees capable of absorbing CO₂. Some CO₂ remains in the atmosphere for hundreds of years, meaning current emissions will continue to affect future climate conditions.

The Fifth Assessment Report estimates that, by the end of this century (2081–2100), relative to the beginning of this century (1986–2005):

1. Global mean surface temperature will rise by 1.0–3.7°C and sea levels by 40–63 cm.
2. The frequency and duration of heat waves will increase.
3. The frequency and intensity of heavy rainfall events will increase.
4. The intensity and/or duration of droughts will increase.
5. Intense tropical cyclone (typhoon) activity will increase.
6. The frequency and/or extent of extreme high sea levels will increase.

In 2015, the Conference of the Parties (COP21) to the UNFCCC adopted the Paris Agreement, which aims to limit global temperature rise to within 2°C above pre-industrial levels, and preferably to 1.5°C.

In 2018, the IPCC published a Special Report on Global Warming of 1.5°C, stating that human-induced warming has already reached approximately 1.0°C. To limit warming to 1.5°C, global net greenhouse gas emissions must reach zero by 2050. Net emissions equal emissions minus removals, achieved through afforestation and various engineering technologies.

Q2.1 Is the following statement correct?
“Climate change is caused by a hole in the atmosphere.”

- Correct
- Incorrect
- Not sure

Q2.2 Is the following statement correct?
“Every time we use coal, oil, or natural gas, we indirectly contribute to climate change.”

- Correct
- Incorrect
- Not sure

Q2.3 Regarding the main cause of warming over the past 50 years, which statement is correct?

- It may have been caused by humans.
- It is impossible that it was caused by humans.

Q2.4 Which of the following phenomena should **not** be caused by humans?

- Rising sea levels
- Increased frequency of strong earthquakes
- Increased frequency of heat waves
- Increased frequency of heavy rainfall
- More severe drought

**Block: Current Carbon Reduction in Taiwan**

Please read the following description and answer the subsequent question.

In 2015, the Taiwanese government enacted the “Greenhouse Gas Reduction and Management Act.” Article 4 specifies that long-term greenhouse gas reduction targets aim to reduce emissions in 2050 to below 50% of the 2005 level. Current policy relies on FIT (Feed-in Tariff), through which power companies purchase renewable energy (wind, solar) at higher prices. Compared with 2005, Taiwan’s greenhouse gas emissions were reduced by 2% in 2020.

Q3 According to current law, by 2050, how much should greenhouse gas emissions be reduced relative to 2005 levels?

- No requirement
- Reduced by 25%
- Reduced by 50%
- Reduced by 100%

**Block: Behavior**

Q4.1 To reduce carbon emissions, are you willing to use public transportation instead of private vehicles?

- Very willing
- Willing
- Unwilling
- Very unwilling

Q4.2 To reduce carbon emissions, are you willing to reduce the use of air conditioning?

- Very willing
- Willing
- Unwilling
- Very unwilling

Q4.2 How often do you walk or bike instead of driving or riding a scooter?

- Always
- Often
- Not often
- Never

**Block: Imagining Future Generations**

Please imagine for one minute that you have a time machine and can travel to Taiwan in the year 2050. You can experience daily life, meet residents, and observe environmental changes. Please write what kind of living environment you observe in 2050.

(Open-ended questions)

**Q5.1** After landing near your home and opening the time machine door, what do you see?

**Q5.2** How does the surrounding environment feel compared to 2021?

**Q5.3** How is the air quality when you take a deep breath?

**Q5.4** How do you feel after interacting with local residents?

**Q5.5** What is the condition of forest plants and animals?

Now, please return by time machine. You are now a representative of future generations. Current carbon emissions will affect future climate change. Next, you will compare carbon reduction policies.

**Block: Policy Choice**

To comply with regulatory requirements to achieve a 50% reduction in carbon emissions by 2050, the government plans to levy a carbon tax. In 2020, the carbon tax rate was set at NTD 420 per metric ton of carbon dioxide, which is equivalent to an increase of NTD 0.21 per kilowatt-hour of electricity and NTD 0.84 per liter of gasoline. The revenue generated from the carbon tax will be redistributed to the public through five different policy schemes.

In the following section, you will be asked to compare five different carbon tax schemes. For each comparison, please choose the option you prefer between the two alternatives presented.

Q6 The table below presents projected outcomes for the year 2050 (relative to the baseline scenario#). Which of the two policy options do you prefer?

| **Impact** | **Option A** | **Option E** |
| --- | --- | --- |
|  | **Carbon Tax** | **No Carbon Tax** |
| Tax revenue | The tax revenue will be used to reduce the VAT rates, household income tax rates, and social security contributions. | No change (Maintain current FIT only) |
| Carbon emissions compared to 2005 | Reduced by 50% | Increased by 41% |
| The annual income of the lowest-income group | Increased by 4.2% | No change |
| GDP | Increased by 4.1% | Reduced by 0.1% |
| Electricity bills per year per person | Increased by NT$11,000 | No change |

- Option A
- Option E

**Notes:**

- **Social security contributions**: Mandatory contributions paid by employers, including labor insurance, labor pension contributions, unemployment insurance, and national health insurance premiums.
- **Feed-in Tariff (FIT)**: A policy under which the government requires electricity companies to purchase renewable energy, such as wind and solar power, at above-market prices.
- **Baseline scenario assumption:** From the present year to 2050, the government does not implement any new carbon reduction policies. Under the existing legal and regulatory framework, markets and resource allocation continue to operate as usual, with an average annual economic growth rate of 1.1%.
- **Lowest income group**: Households nationwide are ranked according to disposable income and divided into five equal-sized groups, each representing 20% of all households. This group refers to the lowest income quintile.
- **Lump-sum transfer**: Tax revenues are redistributed evenly to the citizens on an annual basis.

(… All remaining blocks from AE, EA, BE, EB, … through to the final DC are in the same structure. For detailed descriptions of all policy schemes, please refer to Table 1 in the main text.)

**Block: Other questions**

Q7 To what extent do you agree with the following statements?

| Statement | Strongly disagree | Disagree | Agree | Strongly agree |
| --- | --- | --- | --- | --- |
| In the past year, the air pollution around my residence has been severe. |  |  |  |  |
| In the future, the air pollution around my residence will become more serious. |  |  |  |  |
| I am worried about future climate change phenomena. |  |  |  |  |

Q8 Which natural disasters are you currently concerned about? (Select all that apply)

- Flooding
- Heat waves
- Typhoons
- Drought
- Landslides and debris flows
- Earthquakes
- Other: _____________________________

Q9 Which climate change impacts do you expect to experience in 2050? (Select all that apply)

- Sea level rise
- Increase in average temperature
- Heat waves
- Stronger typhoons
- Stronger heavy rainfall
- Drought
- Other: _____________________________

Q10 Are you willing to make greater efforts to reduce carbon emissions?

- Very willing
- Willing
- Unwilling
- Very unwilling

Q11 If you are unwilling to make greater efforts to reduce carbon emissions, what are the reasons? (Select all that apply)

- I prioritize satisfying my own consumption.
- Individual actions have little effect on reducing carbon emissions.
- Climate change will not affect me.
- I do not believe climate change exists / I do not believe climate change is caused by humans.
- Other: _____________________________

Q12 To what extent do you agree with the following statements?

| Statement | Strongly disagree | Disagree | Agree | Strongly agree |
| --- | --- | --- | --- | --- |
| As inhabitants of Earth, we should contribute to protecting the environment. |  |  |  |  |
| Those who do not practice energy conservation or carbon reduction will be criticized by others. |  |  |  |  |
| Most of my friends and family support spending money to mitigate climate change (e.g., paying higher electricity or fuel prices). |  |  |  |  |

Q13 To what extent do you agree with the following statements?

| Statement | Strongly disagree | Disagree | Agree | Strongly agree |
| --- | --- | --- | --- | --- |
| Tax rates for high-income earners should be increased. |  |  |  |  |
| Paying money (e.g., higher electricity or fuel prices) to reduce carbon emissions is reasonable. |  |  |  |  |
| Stimulating the economy is more important than reducing carbon emissions. |  |  |  |  |

Q14 Do you support the use of a carbon tax to reduce carbon emissions?

- Strongly support
- Support
- Oppose
- Strongly oppose

Q15 For major emitters, what approach do you support for reducing emissions?

- Voluntary corporate reduction
- Administrative regulations on industries or companies
- Subsidies for companies that reduce emissions
- Other: _____________________________

Q16 To what extent do you believe the following statements?

| Statement | Strongly disagree | Disagree | Agree | Strongly agree |
| --- | --- | --- | --- | --- |
| The government will collect taxes fairly. |  |  |  |  |
| The government will use tax revenues efficiently. |  |  |  |  |

Q17 To what extent do you agree with the following statements?

| Statement | Strongly disagree | Disagree | Agree | Strongly agree |
| --- | --- | --- | --- | --- |
| Reducing carbon emissions decreases the level of health or well-being for future generations. |  |  |  |  |
| Actions should be taken to reduce carbon emissions for the sake of future generations. |  |  |  |  |
| Reducing carbon emissions increases the level of health or well-being for myself and my family. |  |  |  |  |

Q18 Does your current residence lie on a hillside?

- Yes
- No

Q19 Does your current residence lie by the sea?

- Yes
- No

Q20 Which county/city do you currently live in?

Q21 What is your biological sex?

- Male
- Female

Q22 In which year were you born?

Q23 What is your personal monthly income?

- NT$20,000 or below
- …
- Above NT$200,000

Q24 What is your household's annual income?

- NT$100,000 or below
- …
- Above NT$2,000,00

Q25 What is your highest level of education (or current school)?

- Elementary school
- Junior high school
- Senior high school / vocational school
- Junior college
- University
- Graduate school or above

Q26 What is your occupation?

- Legislators, supervisors, and managers
- …
- Other

Q27 At your workplace, has there been any discussion regarding responses to potential future carbon tax/fee?

- Yes, planning is in progress
- I have heard about it, but I am not familiar with it
- No discussion at all

Q28 Will your industry (or the industry providing your income) be affected by climate change?

- Positive impact
- Negative impact
- No impact
- Uncertain

Q29 Do you have children to support?

- 0
- 1
- 2
- 3
- 4
- 5 or more

Q30 Are there children aged 12 or younger living in your household?

- Yes
- No

Q31 Do you own a car?

- Yes
- No

Q32 Do you own a motorcycle?

- Yes
- No

Q33 If yes, how frequently do you use a car per week?

- Once or less
- 2–3 times
- 4–5 times
- 6 times or more

Q34 If yes, how frequently do you use a motorcycle per week?

- Once or less
- 2–3 times
- 4–5 times
- 6 times or more

Q35 How frequently do you use air conditioning at home in summer?

- Always
- Often
- Sometimes
- Occasionally
- Rarely
- Almost never
