# Supplementary material for: The impact of imaginary future generations on the preference for carbon tax schemes
Source: PLoS One. 2026 Apr 10;21(4):e0346904. doi: 10.1371/journal.pone.0346904 (PMC13068264; doi:10.1371/journal.pone.0346904)
Supplement: S1 Table — (DOCX) [file pone.0346904.s004.docx]

**Supplementary material**

**S1 Table.** **Robustness analysis of the marginal effects of the Logit model for Schemes A, B, C, and D against Scheme E**

|  | **AE** | **BE** | **CE** | **DE** |
| --- | --- | --- | --- | --- |
| IFGs | 0.123*** (0.030) | 0.113*** (0.029) | 0.110*** (0.030) | 0.121*** (0.031) |
| Age | 0.003* (0.002) | 0.003** (0.002) | 0.002 (0.002) | 0.004* (0.002) |
| Income | 0.005 (0.007) | -0.003 (0.006) | 0.003 (0.007) | -0.003 (0.006) |
| Male | -0.008 (0.033) | 0.0031(0.033) | 0.006 (0.033) | 0.045 (0.034) |
| Living in the Hills | 0.038 (0.070) | 0.089 (0.073) | -0.018 (0.071) | 0.012 (0.076) |
| Living by the Sea | -0.038 (0.041) | -0.015 (0.041) | -0.018 (0.042) | -0.008 (0.045) |
| The Order of the Scheme Attributes | 0.069** (0.030) | 0.067** (0.030) | 0.046 (0.030) | 0.016 (0.032) |
| Number of Cohabiting Children | -0.015 (0.018) | -0.006 (0.018) | 0.000 (0.018) | -0.008 (0.019) |
| College | 0.005 (0.042) | 0.037 (0.042) | 0.064 (0.041) | 0.039 (0.043) |
| **Occupation** (Reference group: Others) | | | | |
| Student | 0.074 (0.065) | 0.147** (0.067) | 0.101 (0.066) | 0.012 (0.066) |
| Homemaker | -0.073 (0.056) | -0.087 (0.055) | -0.007 (0.058) | -0.038 (0.060) |
| Walk | 0.029 (0.022) | 0.017 (0.022) | 0.002 (0.022) | -0.001 (0.022) |
| Air Pollution | 0.048* (0.027) | 0.056** (0.026) | 0.051** (0.026) | 0.020 (0.028) |
| Subjective Norm | 0.033 (0.026) | 0.033 (0.025) | 0.032 (0.026) | 0.072*** (0.026) |
| Government Efficiency | 0.012 (0.020) | -0.011 (0.020) | -0.013 (0.020) | 0.056*** (0.020) |
| Behavioral Control | -0.058** (0.025) | -0.066*** (0.024) | -0.073*** (0.025) | -0.036 (0.027) |
| **Industry Vulnerability** (Reference group: No impact/Unclear) | | | | |
| Positive Impacts | 0.072* (0.044) | 0.037 (0.044) | 0.039 (0.044) | 0.053 (0.047) |
| Negative Impacts | 0.025 (0.043) | 0.006 (0.043) | 0.009 (0.043) | 0.044 (0.047) |
| N | 950 | | | |
| Notes:   1. Standard errors in parentheses * p<.1, ** p<0.05, *** p<0.01. 2. Income: NT$10,000 per unit. 3. Male, living in the hills, living by the sea, and college are dummy variables. 4. The order of the scheme attributes, from top to bottom, is carbon emissions, electricity bills per year per person, and GDP, with the income of the lowest income group at the bottom, coded as 1. The alternative order places carbon emissions at the top, followed by the income of the lowest income group and GDP, with electricity bills per year per person at the bottom, coded as 0. 5. There are three attributes of occupation. Two dummy variables are set up for comparison with the attribute “Others.” 6. There are three attributes of industry vulnerability. Two dummy variables are set up for comparison with the attribute “No impacts or unclear.” | | | | |
